# Inability to Work Fulltime, Prevalence and Associated Factors Among Applicants for Work Disability Benefit

**DOI:** 10.1007/s10926-021-09966-7

**Published:** 2021-03-12

**Authors:** Henk-Jan Boersema, Tialda Hoekstra, Femke Abma, Sandra Brouwer

**Affiliations:** 1 Department of Health Sciences, Community and Occupational Medicine, University Medical Center Groningen, University of Groningen, PO Box 196, 9700 AD Groningen, The Netherlands; 2 Research Center for Insurance Medicine, Amsterdam, The Netherlands; 3 The Dutch Social Security Institute: the Institute for Employee Benefits Schemes (UWV), Amsterdam, The Netherlands

**Keywords:** Chronic disease, Diagnosis, Disability evaluation, Sick leave, Work

## Abstract

*Purpose* Inability to work fulltime is an important outcome in the assessment of workers applying for a disability benefit. However, limited knowledge is available about the prevalence and degree of the inability to work fulltime, the associations between disease-related and socio-demographic factors with inability to work fulltime and whether the prevalence and the associations differ across disease groups. *Methods* Anonymized register data on assessments of workers with residual work capacity (n = 30,177, age 48.8 ± 11.0, 53.9% female) applying for a work disability benefit in 2016 were used. Inability to work fulltime was defined as being able to work less than 8 h per day. *Results* The prevalence of inability to work fulltime was 39.4%, of these 62.5% could work up to 4 h per day. Higher age (OR 1.01, 95% CI 1.01–1.01), female gender (OR 1.45, 95% CI 1.37–1.52), higher education (OR 1.44, 95% CI 1.33–1.55) and multimorbidity (OR 1.06, 95% CI 1.01–1.11) showed higher odds for inability to work fulltime. Highest odds for inability to work fulltime were found for diseases of the blood, neoplasms and diseases of the respiratory system. Within specific disease groups, different associations were identified between disease-related and socio-demographic factors. *Conclusion* The prevalence and degree of inability to work fulltime in work disability benefit assessments is high. Specific chronic diseases are found to have higher odds for inability to work fulltime, and associated factors differ per disease group.

## Introduction

An important aspect of functioning at the level of the whole human being, is the ability of a person to be active in their working life [1, 2]. To determine whether someone is able to work, the concept “work ability” is seen as a standard and a marker for the current ability of a person to perform in a job [3–5]. Work ability reflects the extent to which people can do their job satisfactorily, taking their job demands and their (physical and mental) health into account [6]. Having a chronic disease, associated with activity limitations, can lead to decreased mental and physical functioning [7–9], and therefore threatening work ability and working hours [10–12]. In comparison to healthy workers, workers with a chronic disease work fewer hours and more often part-time due to differences in fatigue and emotional exhaustion [13–15].

In the Netherlands, long-term sick listed workers with a limited ability to work due to a chronic disease may apply for disability benefit to compensate for income loss. As part of the overall disability assessment, the (in)ability to work fulltime, i.e. the number of hours per day and per week the applicant is able to work, is assessed by insurance physicians from the Dutch Social Security Institute, The Institute for Employee Benefits Schemes (UWV). A limitation of working hours due to chronic disease usually results in partial disability in the Netherlands. Also in other countries the assessment of the (in)ability to work fulltime is an aspect of work disability assessment, although there are differences between countries in used definitions and measures to assess this construct [16]. Overall, more research on this topic is warranted, taking into account the huge impact the assessment outcome can have both from societal and individual perspective [17–19].

One knowledge gap is the limited knowledge about the prevalence of the (in)ability to work fulltime. A few studies across Europe reported on the prevalence of inability to work fulltime in their country, i.e. Belgium (2.6%) [20], Finland (2.9%) [21], Denmark (8.4%) [21], the Netherlands (ranging from 17 [22] to 48% [17]), Norway (18.0%) [21] and Sweden (36.3%) [21]. Differences in samples, e.g. type of sick leave (short- or long-term), included disease groups, assessment goals and social security systems, make the reported prevalence difficult to compare. For example the two Dutch studies are not comparable due to inclusion of different types of work disability benefit and timeframe. The first study [22] reported on all outcomes of (long-term) disability assessments for disability pension, for workers, and young handicapped persons in 1 year, while the other study [17] reported on workers with (not permanent) full disability benefit over a 7-year period.

Another knowledge gap is that little is known about socio-demographic and disease-related factors that are associated with inability to work fulltime. Previous studies found that socio-demographic factors, such as age, gender and educational level are associated with having work (dis)ability. For example, older age is assocatiated with a higher risk of having one or more chronic diseases [23] and in particular individuals with a chronic disease are at an increased risk to exit paid employment due to unemployment, disability benefits, and early retirement. [24, 25]. Besides that, women more often suffer from common mental problems (e.g. depressive symptoms) compared to men [26]. Moreover, they work more often part-time [27, 28], and in jobs with low autonomy [29] and high mental work load [30]. These differences may lead to differences in impact of a chronic disease on the ability to work fulltime. In addition to age and gender differences, socio-economic differences may exist. It is known that workers with a low educational level are at a higher risk to exit paid employment compared to workers with a high educational level [24, 25]. These educational differences in disability benefits and unemployment can be explained for, respectively, 40% and 9% primarily due to a higher occurrence of chronic diseases among low educated workers [24].

Type of disease and multimorbidity might also be associated with the prevalence and degree of inability to work fulltime. Chronic diseases like cardiovascular diseases, neoplasms, musculoskeletal disorders, mental diseases and neurological diseases are highly prevalent and disabling diseases among individuals within the working age [10]. The prevalence of multimorbidity, i.e. having at least two chronic diseases, increases along with the ageing process [31, 32] and previous studies found that workers with multimorbidity are at an increased risk of involuntary exit from work, such as unemployment and disability benefits [24, 25].

Against this background, the present study aims to (1) gain insight in the prevalence and degree (number of hours per day able to work) of inability to work fulltime; (2) explore associations between socio-demographic and disease-related factors with inability to work fulltime; and (3) explore whether the prevalence and the associations differs across disease groups in a representative sample of applicants for a work disability benefit.

## Methods

### Institutional Setting

In the Netherlands, social insurance legislation (Work and Income Act; WIA [33]) allows employees to apply for a disability benefit after 2 years of sick leave [34]. Individuals may receive disability benefits for a disease or handicap due to either occupational or non-occupational causes. For the disability benefit assessment, insurance physicians gather information on the medical situation, work- and social situation and functioning of the applicant mainly in an assessment interview and from other sources such as treating- and occupational health physicians. Part of the assessment is a conclusion about an individuals’ (in)ability to work fulltime, reported as the number of hours an applicant can work per day graded in steps of 2 h. Insurance physicians adhere to a guideline with regards to assessing inability to work fulltime; ‘Endurance capacity in work’ [35]. This guideline describes three indications for inability to work fulltime: 1. a lack of energy, resulting in the need for extra daily recovery (hours of rest) consistent with the findings of the insurance physicians and with the nature and severity of the disease, 2. when an increasing number of working hours cause (worsening of) disease symptoms, and 3. reduced availability for work because of necessary treatment. After the medical disability assessment by an insurance physician and assessment of earning capacity by a labor expert of the UWV, individuals can either have a full and permanent work disability, a non-permanent but full work disability, or a permanent and partial work disability. Individuals in the latter group have residual earnings capacity. Individuals with residual capacity are incentivized to continue in paid (part-time) employment at their current employer or enroll in a new, more appropriate (part-time) job, in accordance to their residual work capacity. The income in the original work before sick leave is compared with the income in the work they can perform according to their residual work capacity. The income loss determines the amount of the disability benefit, with a threshold of 35% loss of income. Students, self-employed workers, pensioners and individuals disabled since childhood are not entitled to a WIA-disability benefit. Instead, individuals disabled since childhood can apply for a WAJONG-disability benefit when they turn eighteen (Disablement Assistance Act for Handicapped Young Persons) [36].

### Design and Sample

The study is a cross-sectional register based cohort study among applicants for a long term disability benefit according to the WIA [33], in a year cohort (January 1st to December 31st 2016). The data was provided by the UWV and derived from the register forms completed by the insurance physicians and labor experts at the time of assessment, and anonymized by UWV. For this study, we only included applicants in the analyses with residual work capacity and with complete data on all variables. Approval by a Medical Ethical Committee was not necessary under Dutch law.

### Measures

Socio-demographic data included gender (male/female), age, and educational level. For educational level, three classes were differentiated based on the highest level of completed education: low (primary school, lower vocational education, lower secondary school), middle (intermediate vocational education, upper secondary school), and high (upper vocational education, university).

Insurance physicians use the Dutch Classification of Occupational Health and Social Insurance (CAS) to categorize diagnoses, derived from the International Statistical Classification of Disease and Related Health Problems (ICD-10) [37]. For generalizability, the primary, secondary and tertiary (when available) CAS-diagnoses were recoded to the 22 chapters of the ICD-10 and presented in disease groups. Multimorbidity was defined as having one or more additional diagnosis from a different disease group than the primary diagnosis.

The (in)ability to work fulltime is reported by insurance physicians using five categories: 1 = at least 8 h per day; 2 = no more than 8 h per day; 3 = no more than roughly 6 h per day; 4 = no more than roughly 4 h per day; and 5 = no more than 2 h per day. Being able to work eight or more hours per day (categories 1–2) was considered as normal ability to work fulltime, all else (categories 3–5) was considered as an inability to work fulltime.

### Statistical Methods

First, applicants were described on age, gender, educational level, primary disease groups, multimorbidity, and the degree of (in)ability to work fulltime. Second, differences between applicants with normal ability to work fulltime and applicants with inability to work fulltime were compared using t-tests for continuous data and Chi^2^-tests for categorical and ordinal data. Third, univariable and multivariable logistic regression analyses were performed to study the association of each socio-demographic variable (gender, age, educational level) and disease-related variable (primary disease group and multimorbidity) with the inability to work fulltime (no/yes). Disease group “diseases of the musculoskeletal system and connective tissue” was used as reference category. Fourth, for each of the disease groups population attributable fractions (expressed in percentages) were calculated using Levin’s formula [38, 39] to study the proportional attribution of each disease group to the total number of applicants being assessed with an inability to work fulltime. A high positive percentage for a disease group indicates that the specific disease group has a high attributable fraction to the outcome (being assessed with an inability to work fulltime). A negative percentage indicates a protective fraction to the outcome. Furthermore, univariable and multivariable (adjusted for gender, age, educational level and multimorbidity) logistic regression analyses were performed to study if the primary disease group (no/yes) was associated with the inability to work fulltime, in comparison with all the other applicants in the study sample (not having a disease in that specific disease group as a primary diagnosis). Fifth, multivariable logistic analyses were stratified for each disease group to study the associations between gender, age, educational level and multimorbidity and the inability to work fulltime for each specific disease group. ICD-10 disease groups with a small sample size (n is less than 0.1% of total group) were excluded from the logistic regression analyses.

Analyses were performed using IBM SPSS Statistics version 25. For all analyses a p-level of < 0.05 was considered to indicate statistical significance.

## Results

### Sample Description

We received data from n = 33,179 applicants with residual work capacity from the UWV. In total, 3002 cases were excluded due to missing data on educational level. This group did not differ from the study sample (n = 30,177) on age and on the frequency of applicants in about half of the disease groups. However, the excluded sample consisted of significantly more males (50.1% vs. 46.1%), had less often multimorbidity (36.5% vs. 52.7%), and were more often considered to be able to work fulltime (62.9% vs. 60.6%). The disease groups “no disease”, neoplasms, mental and behavioural disorders, diseases of the nervous system, the eye and adnexa, the circulatory system, congenital malformations and deformations and chromosomal abnormalities, diseases of the musculoskeletal system, the respiratory system, pregnancy and childbirth and the puerperium, and symptoms, signs and abnormal clinical and laboratory findings differed significantly between both groups. Differences ranged from 0.2 to 16.2%, with the largest differences for diseases of the musculoskeletal system (12.2% vs. 28.4%) and mental and behavioural disorders (35.3% vs. 29.5%).

Applicants’ ages in the study sample (n = 30,177) ranged from 18 to 65 years (mean age 48.8 ± 11.0) with 53.9% women, 52.5% finished low education, 33.0% middle education and 14.5% high education. Of the disease groups, the groups with the highest frequencies of primary diagnosis were mental and behavioural disorders (29.5%) followed by diseases of the musculoskeletal system (28.5%). A small majority of the sample had an additional diagnosis in a different disease group (52.7%). The prevalence of inability to work fulltime in the sample was 39.4%. Of the applicants that were assessed with an inability to work fulltime (n = 11,893), the majority (62.5%) were considered to be able to work about 4 h per day (see Table 1 for more detailed information).

**Table 1:** Characteristics of the applicants, and differences between applicants with a normal ability and an inability to work fulltime

	Total group (n = 30,177) n (%)	Ability to work fulltime (n = 18,284) n (%)	Inability to work fulltime (n = 11,893) n (%)	p-value
Age (years) (mean ± sd)	48.8 ± 11.0	48.5 ± 11.1	49.3 ± 10.9	< .001
Female gender	16,258 (53.9%)	9337 (51.1%)	6921 (58.2%)	< .001
Education level				< .001
Low	15,855 (52.5%)	10,407 (56.9%)	5448 (45.8%)	
Middle	9959 (33.0%)	5648 (30.9%)	4311 (36.2%)	
High	4363 (14.5%)	2229 (12.2%)	2134 (17.9%)	
Multimorbidity	15,893 (52.7%)	9415 (51.5%)	6478 (54.5%)	< .001
Disease group				
No disease	13 (0.0%)	13 (0.1%)	–	.004
Infectious and parasitic diseases	142 (0.5%)	55 (0.3%)	87 (0.7%)	< .001
Neoplasms	1908 (6.4%)	580 (3.2%)	1328 (11.2%)	< .001
Diseases of the blood and blood-forming organs	307 (1.0%)	78 (0.4%)	229 (1.9%)	< .001
Endocrine, nutritional and metabolic disorders	490 (1.6%)	272 (1.5%)	218 (1.8%)	.020
Mental and behavioural disorders	8902 (29.5%)	5223 (28.6%)	3679 (30.9%)	< .001
Diseases of the nervous system	1570 (5.2%)	615 (3.4%)	955 (8.0%)	< .001
Diseases of the eye and adnexa	281 (0.9%)	182 (1.0%)	99 (0.8%)	.150
Diseases of the ear and mastoid process	261 (0.9%)	154 (0.8%)	107 (0.9%)	.599
Diseases of the circulatory system	2345 (7.8%)	912 (5.0%)	1433 (12.0%)	< .001
Diseases of the respiratory system	790 (2.6%)	262 (1.4%)	528 (4.4%)	< .001
Diseases of the digestive system	462 (1.5%)	182 (1.0%)	280 (2.4%)	< .001
Diseases of the skin and subcutaneous tissue	134 (0.4%)	101 (0.6%)	33 (0.3%)	< .001
Diseases of the musculoskeletal system and connective tissue	8612 (28.5%)	6854 (37.5%)	1758 (14.8%)	< .001
Diseases of the genitourinary system	275 (0.9%)	95 (0.5%)	180 (1.5%)	< .001
Pregnancy, childbirth and the puerperium	127 (0.4%)	96 (0.5%)	31 (0.3%)	.001
Conditions originating in the perinatal period	–	–	–	
Congenital malformations, deformations and chromosomal abnormalities	137 (0.5%)	67 (0.4%)	70 (0.6%)	.005
Symptoms, signs and abnormal clinical and laboratory findings	1385 (4.6%)	1056 (5.8%)	329 (2.8%)	< .001
Injury, poisoning and certain other consequences of external causes	2010 (6.7)	1473 (8.1%)	537 (4.5%)	< .001
External causes of morbidity and mortality	–	–	–	
Factors influencing health status	26 (0.1%)	14 (0.1%)	12 (0.1%)	.481
Degree of (in)ability to work fulltime				< .001
> 8 h per day	15,370 (50.9%)	15,370 (84.1%)	–	
≤ 8 h per day	2914 (9.7%)	2914 (15.9%)	–	
≤ 6 h per day	2494 (8.3%)	–	2494 (21.0%)	
≤ 4 h per day	7438 (24.6%)	–	7438 (62.5%)	
≤ 2 h per day	1961 (6.5%)	–	1961 (16.5%)	

### Differences Ability and Inability to Work Fulltime

Applicants with a normal ability to work fulltime (n = 18,284, 60.6% of the study sample) were significantly younger (48.5 ± 11.1 vs. 49.3 ± 10.9), more often male (48.9% vs. 41.8%) and had more often a low educational level (56.9% vs. 45.8%) than applicants with an inability to work fulltime (n = 11,893, 39.4% of the study sample). Nearly all disease groups showed significant differences in the frequency of (in)ability to work fulltime between both groups, except for diseases of the eye, diseases of the ear and mastoid process, and factors influencing health status. The five disease groups with the highest frequencies of the primary diagnosis showed the following results: applicants with an ability to work fulltime were significantly more often diagnosed with diseases of the musculoskeletal system (37.5% vs. 14.8%), whereas applicants with an inability to work fulltime were more often diagnosed with neoplasms (11.2% vs. 3.2%), mental and behavioural disorders (30.9% vs. 28.6%), diseases of the nervous system (8.0% vs. 3.4%) and the circulatory system (12.0% vs. 5.0%). Although the majority of both groups were diagnosed with two or more diseases, multimorbidity was significantly more frequent in the applicants with an inability to work fulltime (54.5% vs. 51.5%) (see Table 1 for more detailed information).

### Univariable and Multivariable Regressions Analyses on Inability to Work Fulltime

The uni- and multivariable analyses showed similar significant findings (Table 2). Four ICD-10 disease groups were excluded from the analyses based on a small sample size (less than 0.1% of the total sample): disease groups no disease (n = 13), conditions originating in the perinatal period (n = 0), external causes of morbidity and mortality (n = 0), and factors influencing health status (n = 26). In the final analysis we found higher age (OR 1.01, 95% CI 1.01–1.01), female gender (OR 1.45, 95% CI 1.37–1.52), middle (OR 1.33, 95% CI 1.25–1.40) and high (OR 1.44, 95% CI 1.33–1.55) educational level (compared to low educational level) and multimorbidity (OR 1.06, 95% CI 1.01–1.11) to have a significantly higher risk of the inability to work fulltime. All included disease groups showed higher odds for an inability to work fulltime than the reference disease group “diseases of the musculoskeletal system and connective tissue”.

**Table 2:** Associations of socio-demographic and disease related variables with the inability to work fulltime (univariable and multivariable logistic regression analyses) (n = 30,138)

	Univariable analyses			Multivariable analyses		
	OR	95% CI	p-value	OR	95% CI	p-value
Age (years)	1.01	1.01–1.01	< .001	1.01	1.01–1.01	< .001
Female gender	1.33	1.27–1.40	< .001	1.45	1.37–1.52	< .001
Education level						
Low (ref)	–	–		–	–	
Middle	1.46	1.39–1.54	< .001	1.33	1.25–1.40	< .001
High	1.83	1.71–1.96	< .001	1.44	1.33–1.55	< .001
Multimorbidity	1.13	1.08–1.18	< .001	1.06	1.01–1.11	0.031
Disease group						
Infectious and parasitic diseases	6.16	4.37–8.66	< .001	6.10	4.32–8.61	< .001
Neoplasms	8.28	7.40–9.26	< .001	8.12	7.26–9.08	< .001
Diseases of the blood and blood-forming organs	11.42	8.79–14.85	< .001	10.91	8.37–14.21	< .001
Endocrine, nutritional and metabolic disorders	3.12	2.59–3.76	< .001	3.06	2.54–3.70	< .001
Mental and behavioural disorders	2.74	2.56–2.93	< .001	2.70	2.52–2.90	< .001
Diseases of the nervous system	6.04	5.39–6.77	< .001	5.83	5.19–6.54	< .001
Diseases of the eye and adnexa	2.12	1.65–2.72	< .001	2.01	1.56–2.58	< .001
Diseases of the ear and mastoid process	2.70	2.10–3.48	< .001	2.50	1.94–3.23	< .001
Diseases of the circulatory system	6.11	5.54–6.75	< .001	6.37	5.77–7.05	< .001
Diseases of the respiratory system	7.84	6.70–9.18	< .001	8.11	6.92–9.50	< .001
Diseases of the digestive system	6.00	4.93–7.27	< .001	5.87	4.83–7.14	< .001
Diseases of the skin and subcutaneous tissue	1.27	0.86–1.89	.235	1.30	0.87–1.93	.209
Diseases of the musculoskeletal system and connective tissue (ref)	–	–	–	–	–	–
Diseases of the genitourinary system	7.37	5.72–9.51	< .001	7.29	5.64–9.41	< .001
Pregnancy, childbirth and the puerperium	1.26	0.84–1.89	.273	1.09	0.72–1.65	.674
Congenital malformations, deformations and chromosomal abnormalities	4.07	2.90–5.71	< .001	4.09	2.90–5.76	< .001
Symptoms, signs and abnormal clinical and laboratory findings	1.55	1.37–1.76	< .001	1.20	1.05–1.37	.009
Injury, poisoning and certain other consequences of external causes	1.42	1.27–1.59	< .001	1.46	1.30–1.63	< .001

*OR* odds ratio, *CI* confidence interval, *ref* reference group

### Associations of Each Included Disease Group with the Inability to Work Fulltime

For each of the included disease groups the population attributable fraction for being assessed with inability to work fulltime, and the association with the inability to work fulltime was studied and compared to the all the other applicants in the study sample not having that specific disease group as the primary diagnosis.

Disease groups with the highest population attributable fractions were neoplasms (5.2%) and diseases of the circulatory system (4.6%). Whereas diseases of the musculoskeletal system showed the lowest population attributable fraction (− 19.3%) (Table 3).

**Table 3:** Population attributable fractions and associations of each disease group with the inability to work fulltime (univariable and multivariable logistic regression analyses, adjusted for gender, age, educational level and multimorbidity) (n = 30,138)

	PAF	Univariable analyses			Multivariable analyses		
	%	OR	95% CI	p-value	OR	95% CI	p-value
Infectious and parasitic diseases	0.26	2.44	1.74–3.43	< .001	2.40	1.70–3.37	< .001
Neoplasms	5.17	3.53	3.19–3.91	< .001	3.18	2.87–3.53	< .001
Diseases of the blood and blood-forming organs	0.92	4.58	3.54–5.93	< .001	4.35	3.36–5.65	< .001
Endocrine, nutritional and metabolic disorders	0.21	1.24	1.03–1.48	.021	1.20	1.00–1.44	.051
Mental and behavioural disorders	2.03	1.12	1.07–1.18	< .001	1.13	1.07–1.19	< .001
Diseases of the nervous system	2.98	2.51	2.26–2.78	< .001	2.42	2.18–2.69	< .001
Diseases of the eye and adnexa	− 0.10	0.84	0.65–1.07	.150	0.77	0.60–0.98	.037
Diseases of the ear and mastoid process	0.03	1.07	0.83–1.37	.599	0.95	0.74–1.22	.705
Diseases of the circulatory system	4.64	2.61	2.39–2.85	< .001	2.75	2.52–3.01	< .001
Diseases of the respiratory system	1.87	3.20	2.75–3.71	< .001	3.34	2.87–3.89	< .001
Diseases of the digestive system	0.84	2.40	1.99–2.89	< .001	2.83	1.97–2.88	< .001
Diseases of the skin and subcutaneous tissue	− 0.17	0.50	0.34–0.74	.001	0.52	0.35–0.77	.001
Diseases of the musculoskeletal system and connective tissue	− 19.29	0.29	0.27–0.31	< .001	0.29	0.27–0.30	< .001
Diseases of the genitourinary system	0.61	2.94	2.29–3.78	< .001	2.87	2.23–3.69	< .001
Pregnancy, childbirth and the puerperium	− 0.16	0.50	0.33–0.74	.001	0.46	0.31–0.69	< .001
Congenital malformations, deformations and chromosomal abnormalities	0.14	1.61	1.15–2.25	.005	1.64	1.17–2.30	.004
Symptoms, signs and abnormal clinical and laboratory findings	− 1.91	0.60	0.53–0.67	< .001	0.60	0.53–0.67	< .001
Injury, poisoning and certain other consequences of external causes	− 2.30	0.54	0.49–0.60	< .001	0.56	0.50–0.62	< .001

*PAF* population attributable fraction, *OR* odds ratio, *CI* confidence interval

Univariable analyses showed significantly higher odds ratios for the inability to work fulltime for infectious and parasitic diseases, neoplasms, diseases of the blood and blood-forming organs, endocrine and nutritional and metabolic disorders, mental and behavioural disorders, diseases of the nervous system, the circulatory system, the respiratory system, the digestive system, the genitourinary system, and congenital malformations when compared to the applicants having a disorder of another disease group as the primary disorder. Applicants with diseases of the skin and subcutaneous tissue, the musculoskeletal system, pregnancy, symptoms and signs and abnormal clinical and laboratory findings, and injury and poisoning and certain other consequences of external causes had significantly lower odds ratios. Diseases of the eye and of the ear were not significantly associated with the inability to work fulltime (for odds ratio’s see Table 3).

When adjusted for gender, age, educational level and multimorbidity, multivariable analyses showed similar results, except for endocrine disorders, which was not significantly associated anymore, and applicants with diseases of the eye were significantly less likely to have an inability to work fulltime (Table 3). The disease groups with the highest odds ratios for an inability to work fulltime in the multivariable analyses were diseases of the blood and blood forming organs (OR 4.35, 95% CI 3.36–5.65), neoplasms (OR 3.18, 95% CI 2.87–3.53), and diseases of the respiratory system (OR 3.34, 95% CI 2.87–3.89). The disease groups with the lowest odds for an inability to work fulltime were diseases of the musculoskeletal system and connective tissue (OR 0.29, 95% CI 0.27–0.30), pregnancy, childbirth and the puerperium (OR 0.46, 95% CI 0.31–0.69), and symptoms, signs and abnormal clinical and laboratory findings (OR 0.60, 95% CI 0.53–0.67) (Table 3).

### Associations with the Inability to Work Fulltime Within Each Disease Group

Gender was associated with the inability to work fulltime for 11 disease groups. Women had in ten out of these 11 disease groups higher odds on having an inability to work fulltime compared to men, except for diseases of the genitourinary system. Higher age showed an increased risk to have an inability to work fulltime for the disease groups neoplasms, mental and behavioural disorders, diseases of the respiratory system, musculoskeletal system, and genitourinary system. Educational level was associated with seven disease groups: diseases of the nervous system, the eye, the circulatory system, the musculoskeletal system, pregnancy, symptoms, signs and abnormal clinical and laboratory findings, and injury. For these disease groups (except for diseases of the eye and pregnancy), high and middle educational levels showed significantly higher odds for an inability to work fulltime compared to a low educational level.

Multimorbidity showed higher risk of inability to work fulltime within four disease groups (diseases of the skin, musculoskeletal system, symptoms, signs and abnormal clinical and laboratory findings, and injury), and lower risk within five disease groups (diseases of the blood, nervous system, circulatory system, respiratory system and genitourinary system) (Table 4).

**Table 4:** Associations of gender, age, educational level and multimorbidity with the inability to work fulltime stratified for each disease group (multivariable logistic regression analyses, n = 30,138)

	Gender (male = ref) OR (95% CI)	Age OR (95% CI)	Educational level (low = ref)		Multimorbidity OR (95% CI)
			Middle OR (95% CI)	High OR (95% CI)	
Infectious and parasitic diseases	1.44 (0.70–2.95)	1.00 (0.96–1.03)	0.70 (0.33–1.51)	0.61 (0.23–1.59)	0.96 (0.48–1.93)
Neoplasms	1.31 (1.07–1.61)*	1.02 (1.01–1.03)**	1.05 (0.84–1.31)	1.25 (0.96–1.64)	0.83 (0.68–1.01)
Diseases of the blood and blood-forming organs	1.78 (1.04–3.07)*	1.02 (1.00–1.05)	0.62 (0.34–1.13)	0.79 (0.38–1.64)	0.56 (0.32–0.98)*
Endocrine, nutritional and metabolic disorders	1.57 (1.08–2.29)*	1.00 (0.98–1.02)	1.32 (0.88–1.98)	1.36 (0.79–2.35)	0.95 (0.60–1.52)
Mental and behavioural disorders	1.52 (1.39–1.66)**	1.00 (1.00–1.01)*	1.05 (0.95–1.16)	0.92 (0.82–1.03)	0.95 (0.87–1.04)
Diseases of the nervous system	1.14 (0.91–1.42)	1.00 (0.99–1.01)	2.05 (1.63–2.59)**	2.69 (1.98–3.66)**	0.64 (0.52–0.79)**
Diseases of the eye and adnexa	2.61 (1.54–4.41)**	0.98 (0.85–1.00)	1.37 (0.77–2.42)	2.43 (1.16–5.08)*	1.22 (0.71–2.09)
Diseases of the ear and mastoid process	1.21 (0.73–2.00)	1.01 (0.98–1.04)	1.59 (0.89–2.83)	1.02 (0.52–1.97)	1.44 (0.85–2.44)
Diseases of the circulatory system	1.62 (1.34–1.94)**	1.01 (1.00–1.02)	1.23 (1.02–1.48)*	1.38 (1.05–1.82)*	0.79 (0.67–0.95)*
Diseases of the respiratory system	2.04 (1.49–2.79)**	1.03 (1.01–1.05)*	1.32 (0.92–1.90)	1.82 (0.96–3.43)	0.64 (0.46–0.91)*
Diseases of the digestive system	1.58 (1.07–2.35)*	1.01 (0.99–1.02)	1.17 (0.78–1.77)	1.72 (0.92–3.21)	0.69 (0.45–1.04)
Diseases of the skin and subcutaneous tissue	0.81 (0.36–1.82)	1.00 (0.97–1.04)	1.28 (0.50–3.32)	1.58 (0.78–5.27)	2.88 (1.08–7.70)*
Diseases of the musculoskeletal system and connective tissue	1.55 (1.38–1.73)**	1.01 (1.01–1.02)**	1.60 (1.42–1.80)**	2.90 (2.39–3.52)**	1.66 (1.49–1.85)**
Diseases of the genitourinary system	0.51 (0.30–0.88)*	1.03 (1.00–1.06)*	1.22 (0.68–2.19)	0.99 (0.47–2.09)	0.47 (0.26–0.85)*
Pregnancy, childbirth and the puerperium	n.a.	1.05 (0.96–1.14)	2.25 (0.72–7.00)	4.11 (1.18–14.33)*	1.20 (0.51–2.87)
Congenital malformations, deformations and chromosomal abnormalities	1.10 (0.51–2.35)	1.03 (1.00–1.06)	1.93 (0.89–4.20)	1.51 (0.53–4.30)	1.37 (0.61–3.07)
Symptoms, signs and abnormal clinical and laboratory findings	1.11 (0.86–1.44)	1.01 (1.00–1.02)	1.72 (1.30–2.28)**	1.76 (1.22–2.55)*	1.37 (1.04–1.81)*
Injury, poisoning and certain other consequences of external causes	1.26 (1.03–1.55)*	1.01 (1.00–1.02)	1.96 (1.56–2.56)**	2.85 (2.13–3.82)**	1.35 (1.10–1.66)*

*p < .05, **p < .001

*n.a.* not applicable, due to empty cell(s), the variable was left out of the multivariable regression analysis, *OR* odds ratio, *CI* confidence interval

## Discussion

In a large cross-sectional register based study among a year cohort of applicants assessed for a long-term work disability benefit, the prevalence of inability to work fulltime was 39.4%. Regarding the degree of inability to work fulltime, the number of applicants who could work up to 4 h per day was approximately three times higher in comparison with applicants who could work up to 2 or 6 h per day. In the total sample, including all disease groups, associated factors for inability to work fulltime were higher age, female gender, higher education and multimorbidity. Applicants with diseases of the blood, the respiratory system, neoplasms and diseases of the genitourinary and circulatory system had higher odds for being assessed with inability to work fulltime, while applicants with diseases of the musculoskeletal system, pregnancy and diseases of the skin and injury had lower odds. Studying the association of age, gender, education level and multimorbidity within specific disease groups compared to all other diseases, showed a varying picture. Within 10 of the disease groups, female gender showed higher odds for inability to work fulltime and within seven of the disease groups higher education had the same but weaker effect. Age showed only small effects, and associations with multimorbidity varied.

The prevalence of inability to work fulltime in our study, 39.4%, is substantial but within the variation found in other Dutch studies, showing prevalences varying between 17 and 48% [17, 22]. The variation between prevalences may be due to differences in included populations. Our sample included applicants, generally 2 years after sick leave, applying for long-term disability benefit (WIA), with all diseases. The two Dutch studies differed on the types of work disability benefit and timeframe. The distribution of the degree of inability to work fulltime is in line with findings of other Dutch studies [17, 22] and in European countries [20, 21]. In Sweden (especially in the period between 1960 and 1990) [40], other Nordic countries [21] and Belgium [20], half time work is a legally accepted degree of limitation in work disability assessment during sick leave. However, these numbers are difficult to translate into other samples and social security systems and therefore results of these studies should be considered in the contexts of the social security systems in the countries in which the studies are performed.

Higher age, female gender, higher education and multimorbidity showed higher risks of inability to work fulltime. Although the odds ratios for age and multimorbidity were not that large (1.01 and 1.06 respectively), the cumulative effect of age and working years is substantial; with increasing age, people suffer more from (and have) more chronic diseases [23, 41]. In line with our findings, previous studies showed that women have a greater risk of negative work outcomes such as sick leave and disability [42]. In our study, higher education has a strong positive association with inability to work fulltime compared to lower and middle educational level. This seems to be in contrast with findings from other studies describing that higher educated workers are better able to adjust their work and are less work disabled than lower educated workers who are considered to be more vulnerable, have more health problems and worse working conditions [43–45]. In search for explanations for this difference, we explored if the higher educated workers in our study sample had more often diseases related with higher odds for inability to work fulltime, however this was not the case (data not shown). The difference might be due to a selection in our sample, as our sample was mostly already 2 years on sick leave, and had 2 years to find suitable working arrangements to continue working. Perhaps the selection of workers who were unable to find suitable work adjustments are those applying for a long term disability benefit. It might also be that higher educated people are better able to describe their experienced limitations, or that the effect of a chronic disease on cognitive functions has a more observable effect in daily functioning compared to lower educated people. Insurance physicians may be more inclined to go along with a consistent and credible story in the assessment of inability to work fulltime. Further research on this interesting finding on the association of educational level and inability to work fulltime is therefore recommended.

Different associations were found for the specific disease groups and the inability to work fulltime. The highest odds were found for diseases of the blood, neoplasms, diseases of the respiratory system (all above OR 3.1) and lowest odds for diseases of the musculoskeletal system, pregnancy and diseases of the skin (OR 0.52 and lower). When looking at the two disease groups including the most applicants, results show that diseases of the musculoskeletal system (28.5% of the total cohort) had the lowest risk for inability to work fulltime (OR 0.29). Whereas being diagnosed with a mental disorder (29.5% of the total cohort), showed a significant increased risk for inability to work fulltime (OR 1.13). Mental disorders include a variety of diseases where some disorders do have an impact on energy levels (e.g. severe depression and schizophrenia), while other disorders more often cause emotional disturbance than a lack of energy and therefore do not have an impact on the inability to work fulltime. Musculoskeletal diseases (with by far the lowest risks for inability to work fulltime) are more likely responsible for physical work limitations (like limited walking and standing and lifting weights because of problems with joints and pain) than inability to work fulltime. The diseases in the groups with high odds of inability (like diseases of the blood, respiratory diseases and neoplasms), are often accountable for energy deficits, for example by reduced exercise tolerance or increased fatigue. This is in line with the guideline ‘Endurance capacity in work’ in the Netherlands [35], but also with earlier research findings in European countries, stating that energy deficit is seen as an important reason to limit the ability to work fulltime [16]. Additionally, some diseases cause limitations in available time to work, for example through part-time psychotherapy in a clinic for mental diseases, or dialysis in kidney disease and thus result in inability to work fulltime. To be able to draw conclusions on which diseases attribute the highest to being assessed with inability to work fulltime, population attributable fractions were calculated. The disease groups with the highest population attributable fraction were neoplasms (5.2%) and diseases of the circulatory system (4.6%). These percentages are relatively low, from which we can conclude that being assessed with an inability to work fulltime is not attributable to one or two specific disease groups. Diseases of the musculoskeletal system, however, showed a highly negative percentage (− 19.3%) indicating being a protective fraction to the outcome. The findings in the present study show that the disease the person has, does seem to be important in terms of their ability to work fulltime, as the association between disease groups and inability to work fulltime varies between disease groups. In addition, there are some diseases associated with long term disability but not with an inability to work fulltime, such as musculoskeletal diseases. These diseases are usually more likely associated with physical work limitations and less likely with energy deficits. Our findings indicate that assessors of inability to work fulltime should be aware that various disease groups have higher odds for inability to work fulltime (i.e. diseases of the blood, neoplasms, diseases of the respiratory system) as well that one of the largest disease groups, diseases of the musculoskeletal system, shows a lower risk of inability to work fulltime in applicants who mostly could not fully resume their original work 2 years after sick leave. Furthermore, the population attributable fractions show that being assessed with inability to work fulltime could not be attributed to one specific disease whereas none of the disease groups showed a high proportion of the outcome. Future studies on the risk of individual diseases on inability to work fulltime could help to identify which applicants are at risk for inability to work fulltime, even earlier than at 2 years after sick leave.

Our finding in the total sample, showing a higher risk for inability to work fulltime for multimorbidity, is in line with previous studies [24, 25]. Our findings in the specific disease groups showed that in those disease groups with low risk of inability to work fulltime (such as diseases of the skin and musculoskeletal diseases) multimorbidity increases the risk of inability to work fulltime. Vice versa, in diseases with higher risk of inability to work fulltime (e.g. diseases of the blood and the nervous, respiratory and genitourinary system) multimorbidity lowered the odds for inability to work fulltime. This latter seems counter intuitive, and was therefore discussed with insurance physicians. Insurance physicians indicated that when assessing applicants with severe diseases it is clear that the impact of that disease itself on work capacity, including inability to work fulltime, is so unambiguous that further exploration of the medical situation is felt unnecessary. In these cases no additional diagnosis are registered, and therefore not in our dataset, because these have no additional value to the outcome of the work disability assessment. Further research on the impact of multimorbidity, including the effect of the number of diagnosis and specific combinations of diagnoses, on inability to work fulltime is therefore recommended.

Our study was a first step towards exploring inability to work fulltime as an outcome of work disability assessment, using register data from work disability assessments according to the UWV. Due to the administrative data source, data was available on diagnosis and certain personal factors. Future studies on inability to work fulltime could be enriched with data from for example assessment reports and questionnaires on therapy, the course of the disease, severity of the disease, and on work and environmental factors to obtain more insight in the position of inability to work fulltime within the biopsychosocial model.

### Strengths and Limitations

In this study, register data of a year cohort of applicants assessed for a long term work disability benefit, covering the entire Dutch population, was used. A strength of this study is the large sample including all assessments, data describing socio-demographics and all diagnoses in a representative sample. Additionally, all comprehensive assessments were carried out by skilled professionals adhering to professional guidelines and assessment methods. A study limitation is that register data was not collected for research purposes and did not contain data on possible determinants such as severity of diseases and treatment, or work and environmental factors. Although the Dutch Social Security System is using a biopsychosocial approach in the work disability assessment, important factors described in this model are lacking in the register data. Unfortunately, we had to exclude 3002 cases because of missing data (only) on educational level, this might have impacted our outcomes, as they had significantly more often a normal ability to work compared to the included sample. Additionally, the cross-sectional design prevents us from drawing conclusions about causal relationships.

## Conclusion

The prevalence of inability to work fulltime in work disability benefits assessment is high: 39.4%. Of these applicants with inability to work fulltime, the majority is assessed as not being able to work over 4 h per day. In the total sample, age, gender, education, multimorbidity and specific disease groups were associated with inability to work fulltime. The risk of inability to work fulltime varies between disease groups, with diseases of the blood, the respiratory system, neoplasms and diseases of the genitourinary and circulatory system showing high odds, and musculoskeletal diseases, the largest group in the sample, showing low odds. Within specific disease groups, compared to all other disease groups, different associations were identified for age, gender, education and multimorbidity, with female and higher educated applicants having higher odds, age having no effect and the effect of multimorbidity differing across disease groups. The findings of this study can contribute to a more evidence based assessment of inability to work fulltime in disability claim assessments, providing insight into which workers with chronic diseases are at risk for inability to work fulltime and can contribute to the development of interventions for work adjustments for workers with inability to work fulltime.

## Data Availability

The data that support the findings of this study were made available from UWV. However, restrictions apply to the availability of these data, which were used under license for the current study, and so are not publicly available. Data are however available from the authors upon reasonable request and with permission of UWV.
